# Cell-Surface GRP78-Targeted Chimeric Antigen Receptor T Cells Eliminate Lung Cancer Tumor Xenografts

**DOI:** 10.3390/ijms25010564

**Published:** 2024-01-01

**Authors:** Shijie Wang, Wenwen Wei, Yuncang Yuan, Jing Guo, Dandan Liang, Xudong Zhao

**Affiliations:** Department of Targeting Therapy & Immunology and Laboratory of Animal Tumor Models, Cancer Center and State Key Laboratory of Respiratory Health and Multimorbidity and Frontiers Science Center for Disease-Related Molecular Network, West China Hospital, Sichuan University, Chengdu 610041, China; wangshijie@wchscu.cn (S.W.); weiwenwen@wchscu.cn (W.W.); yuanyuncang@wchscu.cn (Y.Y.); guojing@wchscu.cn (J.G.); liangdandan@wchscu.cn (D.L.)

**Keywords:** CAR-T cells, csGRP78, immunotherapy, lung cancer

## Abstract

Lung cancer is one of the most common and intractable malignancies. It is associated with low survival rates despite existing treatments, indicating that new and more effective therapies are urgently needed such as the chimeric antigen receptor-T (CAR-T) cell immunotherapy. The cell-surface glucose-regulated protein 78 (csGRP78) is expressed in various hematological malignancies and solid tumor cells including lung cancer in response to cancer-related endoplasmic reticulum stress, while GRP78 is restricted to inside the normal cells. Here, we detected the prominent expression of csGRP78 in both lung cancer cell lines, A549 and H1299, as well as cancer stemlike cells derived from A549 by immunofluorescence. Next, a csGRP78-targeted CAR was constructed, and the transduced CAR-T cells were tested for their potency to kill the two lung cancer cell lines and derived stemlike cells, which was correlated with specific interferon γ release in vitro. Finally, we found that csGRP78 CAR-T cells also efficiently killed both lung cancer cells and cancer stemlike cells, resulting into the elimination of tumor xenografts in vivo, neither with any evidence of relapse after 63 days of tumor clearance nor any detrimental impact on other body organs we examined. Our study reveals the capacity of csGRP78 as a therapeutic target and offers valuable insight into the development of csGRP78 CAR-T cells as potential therapy for lung cancer.

## 1. Introduction

Lung cancer is the most common cause of cancer-related death and the second most common malignancy worldwide [1]. According to global cancer statistics, more than 2 million individuals are estimated to be newly diagnosed with lung cancer annually [2,3], and lung cancer accounts for over 20% of annual cancer deaths, more than the burdens of prostate, breast, and colon cancers combined [4]. Despite significant improvements over the last two decades in treatment strategies, including surgery, chemotherapy, radiotherapy, and especially targeted therapy, the 5-year survival rate of patients remains at approximately 19% [5,6]. Therefore, it is of critical importance that more effective therapeutic strategies are developed for lung carcinoma treatment.

In the last decade, the development and implementation of CAR-T cell therapy to target CD19 expression has been transformative as a new therapeutic approach to effectively treat hematological malignancy [7,8]. Yet, in terms of targeting tumor antigens to treat lung cancer, the development of effective CAR-T cell therapies has been challenging, particularly to identify tumor antigens that are highly expressed, compared to nontumor cells within the lung, and compared with cells in other body organs. Investigations with CAR-T cell approaches that target the epidermal growth factor receptor (EGFR) [9,10], mesothelin (MSLN) [11,12,13], programmed cell death ligand 1 (PD-L1) [14,15], and B7 homolog 3 (B7-H3) [16,17] have so far met with success but remains to be refined. Nevertheless, in recent clinical trials of CAR-T cells targeting the EGFR (NCT01869166) and PD-L1 (NCT03060343) [18], such CAR-T cell therapies have shown safety and partial remission in patients. Other clinical trials that target carcinoembryonic antigen (CEA) CAR-T (NCT04348643) [19] and anti-B7-H3 CAR-T (NCT04842812) therapies [18] are also ongoing. Common challenges faced when developing CAR-T cell therapy in solid tumors, such as in lung cancer, include the heterogeneity in antigen expression, a high genomic instability within solid tumors, as well as tumor cell adaptation. For these three challenges, respectively, low-antigen-expressing tumor cells may escape killing, mutate into more complex genetic variants, or even form antigen-loss variants following immune selection pressure from CAR-T treatment, leading to immune escape [20]. As such, it remains important to continue to further explore tumor targets for CAR-T therapies.

GRP78, also known as immunoglobulin heavy chain binding protein (BiP), has a KDEL motif that acts as a molecular chaperone in the endoplasmic reticulum (ER) and is involved in the proper folding and assembly of proteins, the degradation of misfolded proteins through the proteasome, the coordination of proteins that bind to Ca^2+^ as well as to the ER, and in the activation of transmembrane ER stress sensors [21]. Researchers speculated that under cancer-related stress, as the plasma membrane originates from ER membrane, the ER membrane-spanned subpopulation of GRP78 transduces to the cell surface, interacts with other cell-surface proteins like glycosylphosphatidylinositol-anchored proteins, and preferentially exists as peripheral protein on the plasma membrane, which is never or rarely observed in normal cells [22]. Ultimately, GRP78 becomes the cell-surface GRP78 (csGRP78) displayed on cancer cells.

GRP78 is highly expressed and partially translocated to the cell surface, such as in hematological malignancies and in cells within solid tumors, including lung cancer tumors [23]. This molecular characteristic of GRP78 within cells is due to the poor vascular hyperplasia and high proliferation behaviors of cells within tumors, which altogether induces stress responses in these cells [24]. CsGRP78, as a high-affinity receptor for activated a2-macroglobulin, is postulated to promote the proliferation, survival, and metastasis of prostate cancer cells [25,26,27]. Such cellular effects have so far identified GRP78 as an attractive therapeutic target for prostate tumor cells; however, its targeting of other cancers, including lung cancer, is highly feasible, as outlined in several studies. For example, Ran and colleagues recently described a combination drug delivery approach in which csGRP78 ligand prepared in micelles, together with paclitaxel and temozolomide, was cytotoxic to glioma tumor cells and stem cells, when compared alongside treatment with non-csGRP78 ligand-decorated micelles [28]. In another study, the conjugation of a csGRP78-specific peptide (GIRLRG) was found to increase the circulation of a nanodrug in lung cancer-, pancreatic cancer-, as well as glioma tumor-bearing mice [29]. Moreover, CAR-T cells targeting csGRP78 eliminated acute myeloid leukemia cells and prolonged mice survival [30,31]. Together, these studies suggest the potential therapeutic applications for csGRP78-targeted CAR-T cells to treat liquid as well as solid cancers.

Compared with normal lung tissue, GRP78 is significantly overexpressed at the mRNA and protein levels in lung cancer tissue. The overexpression of GRP78 in lung cancer tissues is closely correlated with the differentiation and development of lung cancer [32,33]. A global profiling analysis of cell surface proteomes has shown that csGRP78 expression is elevated in lung cancer cells, but not on the surfaces of normal lung cells [34]. However, the effects of csGRP78-targeted CAR-T cells against lung cancer have not been demonstrated. Here, we took advantage of the elevated expression of csGRP78 in lung tumor cells to evaluate the antitumor efficacy of csGRP78 CAR-T cells in cell culture studies and in a tumor xenograft model.

## 2. Results

### 2.1. Pep42-BBZ CAR-T Cells Are Cytotoxic to Lung Cancer Cell Lines That Express csGRP78

**Figure 1:**
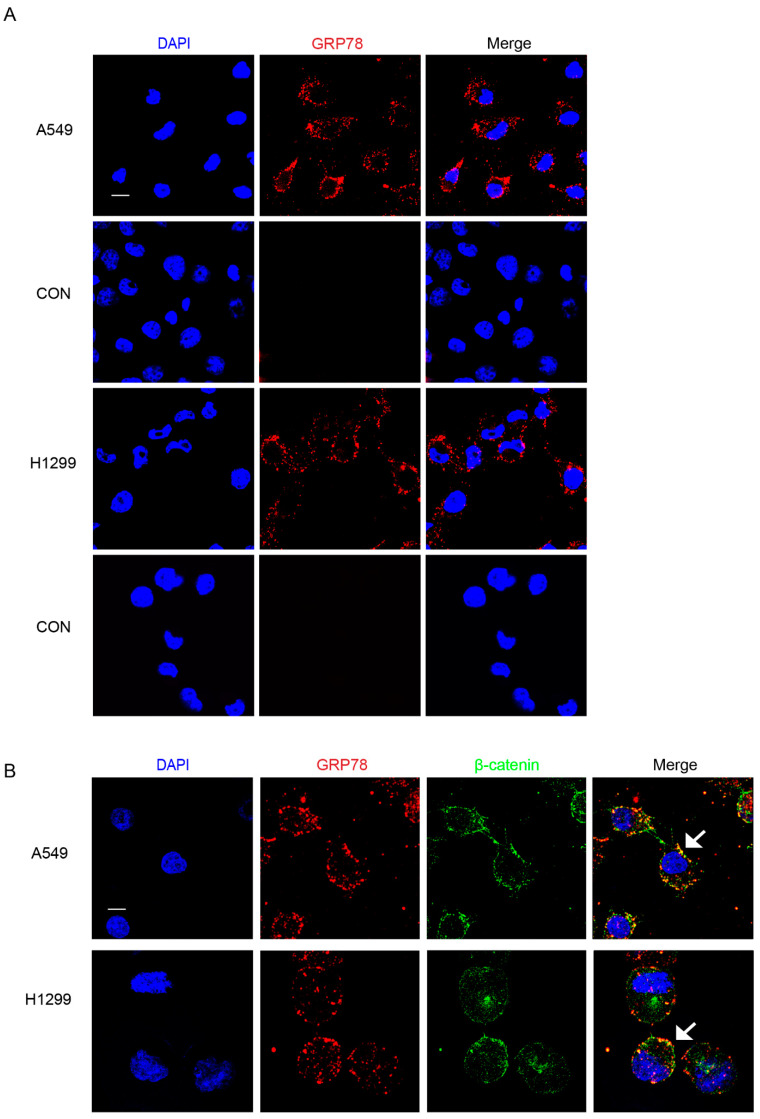
GRP78 is expressed on the surface of human lung cancer cell lines. IF analysis using surface staining protocol on two lung cancer cell lines, A549 and H1299, showing the expression and localization of csGRP78. (**A**) Cells were stained with anti-GRP78 antibody followed by Cy3-conjuncted secondary antibody. Cells only stained with Cy3-conjuncted secondary antibody were used as controls (CON). Scale bar = 20 μm. (**B**) Cells were stained with csGRP78 and β-catenin to show their colocalization. Cy3 labeled csGRP78 and Alexa Flour 488 labeled β-catenin. The colocalization signal turned out to be yellow as indicated by arrows in merging images. Scale bar = 10 μm.

Above all, to better visualize the overall study design and main conclusions, a schematic diagram of this research is displayed (Figure S1). To clarify the cell surface expression of GRP78 on human lung cancer cell lines, we performed immunofluorescence (IF) on two cell lines, A549 and H1299. As shown, we detected prominent csGRP78 signals for both cell lines with the expression percentage of 90% on A549 cells and 89% on H1299 cells (Figure 1A). To more precisely locate csGRP78, stainings of csGRP78 and β-catenin, which is a cell-membrane marker, were performed simultaneously. Both A549 and H1299 cells showed that GRP78 was colocalized with β-catenin on the cell surface (Figure 1B).

**Figure 2:**
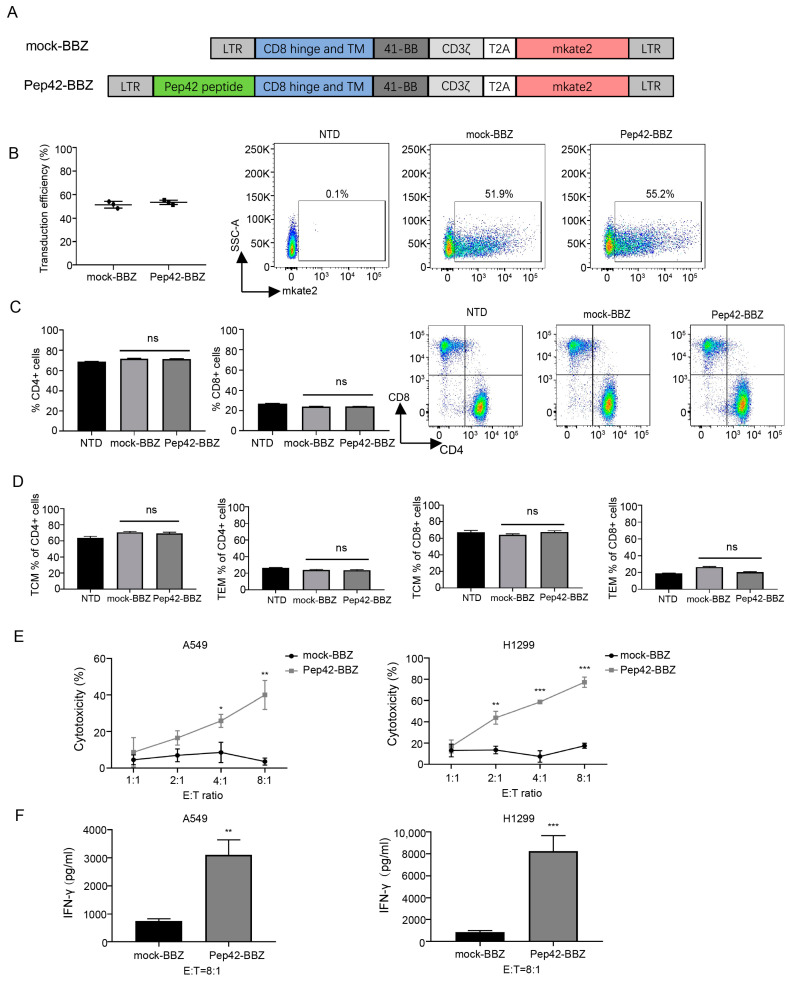
Pep42-BBZ CAR-T cells are cytotoxic to lung cancer cell lines that express csGRP78. (**A**) The structure of the CAR is shown schematically. (**B**) Transduction efficiency of CAR-positive T cells from three groups (nontransduced T cell (NTD), mock-BBZ CAR-T cell, and Pep42-BBZ CAR-T cell) detected by flow cytometry at the extra 96 h culturing post-transduction for 24 h. Left: statistical data from three healthy donors. Right: a set of representative images from one of the three donors. (**C**,**D**) Phenotypic analysis of the three groups of T cells detected by flow cytometry at the extra 96 h of culturing post-transduction for 24 h. Cells were stained with surface markers CD4, CD8, CCR7, and CD45RO. Results were from repeated experiments on T cells from three healthy donors. (**C**) Statistical results and flow cytometry representative images of CD4+ T cells and CD8+ T cells of the total T cells. (**D**) Frequencies of effector memory T cell (TEM, defined by CCR7- CD45RO+) and central memory T cell (TCM, defined by CCR7+ CD45RO+) subpopulations of CD4+ T cells and CD8+ T cells. (**E**) Killing assays between mock-BBZ CAR-T cells or Pep42-BBZ CAR-T cells (effector cells) and A549 or H1299 cell lines (target cells) at gradient ratios of 1:1, 2:1, 4:1, and 8:1, with a coculturing time of 20 h. Results were from repeated experiments on T cells from the three healthy donors and calculated based on killing proportions of NTD cells to the target cells at corresponding coculture ratios. The results are presented as the mean volume ± SD, * *p* < 0.05, ** *p* < 0.01, *** *p* < 0.001. (**F**) IFN-γ release level detection by ELISA. Samples were the supernatant from the coculture system with an E:T ratio of 8:1. The results are presented as the mean volume ± SD, ** *p* < 0.01, *** *p* < 0.001.

Next, we developed a CAR lentiviral vector that included the GRP78-binding peptide Pep42, a 4-1BB sequence, and a CD3ζactivation domain, named Pep42-BBZ, fused with the far-red fluorescence protein mkate2 as a marker by a T2A linker. Mock-BBZ including the same backbone with the absence of the Pep42 peptide was the negative control (Figure 2A). To prepare CAR-T cells, native T cells from three different healthy donors were first incubated with human CD3/CD28 beads for 72 h, following which cells were transduced with either Pep42-BBZ or mock-BBZ lentivirus for another 24 h. To assess CAR expression ratios on T cells, we performed a flow cytometry test at 96 h post-transfection and found that the average transduction efficiency of Pep42-BBZ CAR positive T cells was 53.5 ± 1.8%, which was comparable with that of the mock-BBZ CAR positive T cells of 51.5 ± 2.9% (Figure 2B). Moreover, to observe whether the CAR expression would impact T cell phenotypes, T cell subset markers were stained and analyzed using flow cytometry. Compared with the mock-BBZ CAR-T cell group, no significant difference was displayed in CD4+ T cell populations and in CD8+ T cell populations of total T cells in the Pep42-BBZ CAR-T cell group (Figure 2C). Notably, compared with the mock-BBZ CAR-T cell group, no significant difference was displayed in TEM subpopulations or TCM subpopulations of either CD4+ cells or CD8+ cells in the Pep42-BBZ CAR-T cell group (Figure 2D).

To assess the killing ability of Pep42-BBZ CAR-T cells (effector cells), we next co-cultured these with the target cells (i.e., lung cancer cells) at increasing ratios of E:T = 1:1, 2:1, 4:1, and 8:1. As shown, when compared with the mock-BBZ group, Pep42-BBZ CAR-T cells significantly lysed both of these csGRP78-expressing lung cancer cell lines, A549 and H1299, in a dosage-dependent manner (Figure 2E). Because it is IFN-γ production that dominantly responds for the tumor elimination by CAR T cells, to further confirm the specificity of the effect, supernatants from infection ratios of 8:1 from each of these conditions were collected to measure the IFN-γ release by ELISA. As shown, levels of IFN-γ were significantly higher in target cells incubated with Pep42-BBZ CAR-T cells, compared with target cells incubated with mock-BBZ CAR-T cells (Figure 2F). These data suggest that it is the Pep42 sequence in the CAR structure recognized by the tumor antigen csGRP78 that contributes CAR-T cells to kill these two lung cancer cell lines, rather than differentiated state distinctions of T cell subtypes due to CAR expression.

### 2.2. Pep42-BBZ CAR-T Cells Are Cytotoxic to CSCs In Vitro

**Figure 3:**
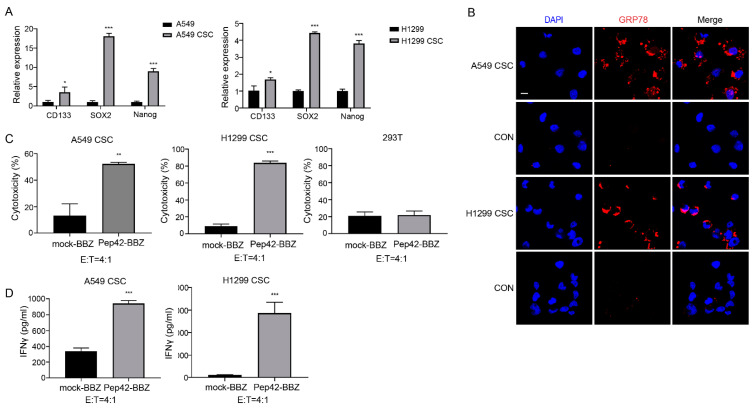
Pep42-BBZ CAR-T cells are cytotoxic to A549 CSCs in vitro. (**A**) Real-time PCR test of three CSC markers’ (CD133, SOX2, and Nanog) expressions on A549 VS A549 CSC and H1299 VS H1299 CSC. 18s was used as a reference gene. The results are presented as the mean volume ± SD, * *p* < 0.05, *** *p* < 0.001. (**B**) IF analysis on A549 CSCs and H1299 CSC showed csGRP78 expression. The surface staining was performed on cells with the GRP78 antibody followed by the Cy3-conjuncted secondary antibody. Cells only stained with the Cy3-conjuncted secondary antibody were used as controls. Scale bar = 10 μm. (**C**) Cytotoxicity of CAR-T cells towards A549 CSCs and H1299 CSCs. 293T cells were a control group of normal cells without cancer. Target cells were cultured on laminin-coating coverslips 36 h ahead of coculturing. Effector cells (CAR-T cells) and target cells (A549 CSCs and H1299 CSCs) were cocultured for 20 h at an E:T ratio of 4:1. Results were from three repeated experiments and calculated based on the killing proportions of NTD cells to the target cells. Results are presented as the mean volume ± SD, ** *p* < 0.01. (**D**) IFN-γ release detection by ELISA. Samples were the supernatant from the coculture system. The results are presented as the mean volume ± SD, *** *p* < 0.001.

CSCs are defined as a subpopulation of cells originating from the primary tumor, displaying stemlike features, and are implicated in tumor initiation, progression, and relapse, as well as metastatic disease [35]. To explore the expression of csGRP78 on CSCs, we generated cultures of A549-derived stemlike spheres and H1299-derived stemlike spheres, named A549 CSCs and H1299 CSCs, respectively, by exposure of cells to classical stem-cell-induced medium. To confirm their stemness, we profiled the expression of three stem-cell markers for lung CSCs, CD133, SOX2, and Nanog [36], by qRT-PCR, as shown (Figure 3A). We found that levels of CD133, SOX2, and Nanog were all significantly increased, when levels for these three markers were quantified in A549 cells or H1299 cells that were not cultured in a CSC-permissive medium. An IF test showed GRP78 was strongly expressed on the surface of A549 CSCs and H1299 CSCs (Figure 3B). Next, we investigated the cytotoxicity of Pep42-BBZ CAR-T cells upon exposure to CSCs. As shown, Pep42-BBZ CAR-T cells killed approximately 50% of A549 CSCs and 80% of H1299 CSCs at the ratio of E:T = 4:1 (Figure 3C). This ratio’s selection referenced the killing results of CAR-T cells to A549 and H1299 cell lines. The killing effects on 293T cells were as a normal control. Moreover, the IFN-γ level in the supernatant was significantly elevated in the Pep42-BBZ CAR-T cells’ coculture system, compared to mock-BBZ exposed cells (Figure 3D). Taken together, Pep42-BBZ CAR-T cells are capable of effectively and specifically killing the two lung cancer CSCs by recognizing csGRP78.

### 2.3. Pep42-BBZ CAR-T Cells Eliminate A549 Tumor Xenografts

**Figure 4:**
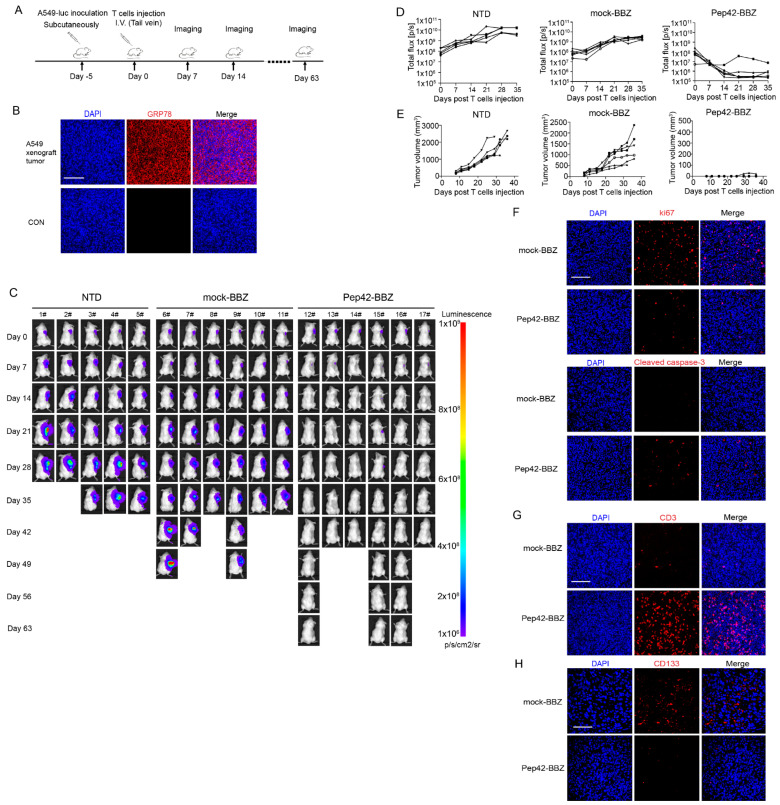
Pep42-BBZ CAR-T cells eliminate A549 tumor cells and CSCs. (**A**) The schematic of the tumor xenograft experiment design. NCG mice were inoculated subcutaneously with 5 × 10^6^ A549-luc cells. Five days later, mice were randomly divided into three groups: NTD (*n* = 5), mock-BBZ (*n* = 6), and Pep42-BBZ (*n* = 6). T cells (1 × 10^7^ per mouse), as indicated, were injected into the tail veins. In vivo imaging software (IVIS) imaging was performed once a week. (**B**) IF staining representatives of csGRP78 expression in A549 tumor xenograft sections. Five days after A549 cells’ inoculation, tumor sections were prepared for csGRP78 detection. Samples only stained with Cy3-conjuncted secondary antibody were used as negative controls. (**C**) Images of tumor bioluminescence by IVIS. (**D**) Tumor bioluminescence’s progressing curves of the three groups. (**E**) Tumor volume’s progressing curves of the three groups. The size was measured twice a week from day 8. (**F**) Representative IF images of ki67 and cleaved caspase-3 staining in tumor sections. Mock-BBZ and Pep42-BBZ CAR-T cells were injected into the tail veins of tumor-bearing mice (*n* = 3 per group). After 5 days, the tumors were extracted, prepared into paraffin sections, and stained with CD3ζ antibody followed by Cy3-conjunctated secondary antibody. Scale bar = 100 μm. (**G**) Representative IF images of T cell staining in tumor sections. (**H**) Representative IF images of CD133 staining in tumor sections. Samples were prepared the same as in **F** and stained with CD3ζ antibody followed by Cy3-conjunctated secondary antibody. Scale bar = 100 μm.

To investigate the antitumor activity of csGRP78 CAR-T cells in vivo, we generated a lung cancer xenograft model by subcutaneous injection in NCG mice with A549 cells stably expressing luciferase reporter gene into their left flank (Figure 4A). Five days later, tumor tissues were extracted and IF staining was performed to confirm csGRP78 expression within tumor cells (Figure 4B). Next, mice were divided into three groups, with mice from each group injected with NTD, mock-BBZ, or Pep42-BBZ CAR-T cells. The bioluminescence intensity profiles for each mouse were then measured once a week until the tumor volume reached ~2000 mm^3^. Compared with the NTD or mock-BBZ CAR-T groups, tumors in mice treated with Pep42-BBZ CAR-T cells were significantly decreased in size and were not detectable by our scans in five out of six of these mice, by day 14. Three of these mice from the Pep42-BBZ CAR-T cell treatment group were additionally kept continuously for observation until day 63, during which no relapse in tumor growth was observed (Figure 4C–E). Moreover, stainings on ki67 in tumor tissue sections revealed an attenuated tumor cell proliferation in the Pep42-BBZ CAR-T cell treatment group, which was consistent with the enhanced tumor cell apoptosis marked by cleaved caspase-3, compared with the mock-BBZ CAR-T group (Figure 4F).

Next, we demonstrated the infiltration of Pep42-BBZ CAR-T cells in injected mice by performing immunostaining using an CD3ζ antibody on sections of tissue from the site of the tumor; 5 days after, the CAR-T cells were injected into tumor-bearing mice. Our IF results show that Pep42-BBZ CAR-T cells infiltrated into the tumors, while mock-BBZ CAR-T cell-injected mice did not show this pattern of staining (Figure 4G). Therefore, this is the direct evidence of our CAR-T cells having a killing ability within tumors. Moreover, we also examined the killing ability of Pep42-BBZ CAR-T cells to CSCs by detecting the lung cancer stem marker CD133 within tumors. Tumor sections from both groups demonstrated that CD133 was highly expressed in the mock-BBZ CAR-T group while slightly expressed in the Pep42-BBZ CAR-T group (Figure 4H). These data suggested that Pep42-BBZ CAR-T cells could kill both tumor cells and CSCs within A549-derived tumor xenografts and ultimately eliminate tumors.

### 2.4. Pep42-BBZ CAR-T Cells Are Safe to Normal Organs

**Figure 5:**
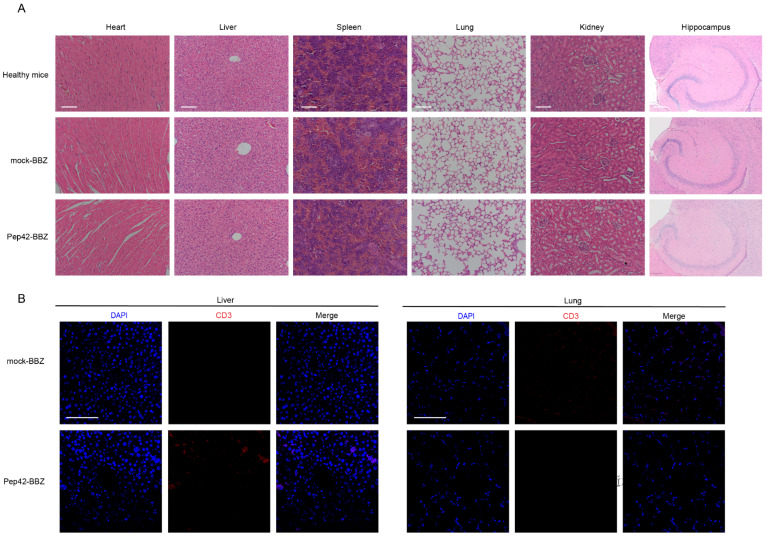
Pep42-BBZ CAR-T cells are safe to normal organs. (**A**) Representative images of H&E staining on the heart, liver, spleen, lung, kidney, and brain sections from healthy mice, in the mock-BBZ group and Pep42-BBZ CAR-T group. Two groups of CAR-T cells were injected into the tail veins of tumor-bearing mice (*n* = 3). After 5 days, six main organs were extracted and prepared into paraffin sections. Scale bars of the hearts, livers, spleens, lungs, and kidneys = 100 μm. Scale bar of the hippocampus = 200 μm. (**B**) Representative IF images of T cell staining on the livers and lungs. Sections were stained with CD3ζ antibody followed by Cy3-conjunctated secondary antibody. Scale bar = 100 μm.

Further, we investigated whether injected T cells might colonize in other organs such as the heart, liver, spleen, lung, kidney, and brain to cause tissue injury. We dissected these tissues and processed them for H&E staining to visualize their general cell and tissue characteristics. Compared with healthy mice (Figure 5A), neither of the properties of these organs from the mock-BBZ CAR-T cell group nor from the Pep42-BBZ CAR-T cell group were abnormal. Finally, because lung and liver are organs with abundant blood supply, we observed only very few CD3ζ immunoreactivity in some cells within the blood vessel cavities of sectioned lung and liver (Figure 5B) in the Pep42-BBZ CAR-T cell treatment group. Thus, Pep42-BBZ CAR-T cells were recruited specifically to target tumor cells while sparing normal tissues.

## 3. Discussion

It has been recognized that csGRP78 is a marker of tumor cells that may be informative as a target for anticancer treatments. While recent studies of CAR-T cells targeting csGRP78 have shown great promise against myeloid cells [30,31], glioblastoma cells [37], and pancreatic cancer cells [38], its cytotoxicity against lung cancer cells has not yet been reported. Here, we confirmed that csGRP78 was immunodetectable and localized on the surface of the two lung cancer cell lines as well as CSCs derived from them. Moreover, we showed that Pep42-BBZ CAR-T cells could efficiently kill both lung cancer cells and CSCs by targeting csGRP78 with a specific IFN-γ release in vitro. Furthermore, these results were mirrored in vivo based on the elimination of experimental lung cancer xenografts derived from injections of A549 cells without relapse by Pep42 CAR-T cells, and without detrimental impact on nontumor tissues.

Lung cancer can be effectively treated when diagnosed in its early stages. However, it is often only detected at symptomatic stages of the disease, where effective treatments are still lacking, and poor survival is documented in over 70% of patients [39]. The viability of CAR-T cell therapies to treat lung cancer is currently under intense investigation, as reflected in current preclinical and clinical trials for this approach. Yet, despite the efficient cytotoxic actions of CAR-T cells to kill cancer cell lines in vitro, its effects in vivo currently are not associated with satisfactory outcomes. For example, in a study of MSLN-directed CAR-T cell therapies for patients with metastatic lung cancer (NCT02414269), of 15 participants enrolled, only one patient had stable disease 3.5 months after treatment, while all others had progressive disease [19]. In another example, Liu and coworkers generated potent cytotoxic anti-PD-L1 CAR-T cells that failed to completely eliminate PD-L1^high^ NSCLC tumor xenografts in mice [15]. These observations show that CAR-T cell therapies remain to be better developed as an approach to effectively treat solid tumors in vivo.

The development of effective CAR-T cell therapies for the treatment of solid tumors has been facing significant issues. Among these, choosing the tumor-specific antigen plays the most important role. As it is not easy to find, searching for the tumor-associated antigen provides an alternative path. In normal cells, GRP78 is primarily localized in the ER lumen as a chaperone protein. However, in response to ER stress from tumor cells, GRP78 has a relocation bias from the ER to the cell surface. In addition, in the case of normal tissues, this elevation of ER stress also signals apoptosis, which leads to a rapid elimination of these cells [40,41]. Based on this line of reasoning, the overlapping expression of csGRP78 between the tumor cells and healthy cells is unlikely to exist. Here, we performed surface immunofluorescence studies to confirm that csGRP78 was prominently expressed on A549 and H1299 lung carcinoma cell lines and xenografts of A549-derived tumors, which is consistent with previous findings [42]. Further, we used β-catenin, a cell-surface marker, to locate GRP78 more precisely on the cell surface of the lung cancer cells. Taken together, csGRP78 has an ideal potency to be targeted by CAR-T cells.

CSCs are thought to be cancer-initiating cells and contribute to the formation of tumor recurrences, metastases, and therapeutic resistance, owing to their high self-renewal capacity [43]. As a result, CSCs are also considered to be important therapeutic targets. For example, glioblastoma stem cells could be killed by CD133-targeted CAR-T cells and the survival of tumor-bearing mice was prolonged [43]. The CSCs of renal cell carcinoma (RCC) and osteosarcoma could be specifically killed by DNAJB8-targeted CAR-T cells and tumors were significantly suppressed in human RCC xenografts [44]. Recent studies revealed the positive influence of GRP78 on the epithelial–mesenchymal transition and CSCs [45,46,47]. GRP78 has been documented to localize to the surface of stem cells and shown to play an important role in reprogramming, promoting pluripotent activity, and in enhancing the expression of stemness genes [48]. In our study, we confirmed that GRP78 was immunodetectable on the cell surface of A549-derived stemlike cells, and that A549 CSCs were susceptible to killing by Pep42 CAR-T cells in vitro. Also, IF stainings on tumor sections from the mock-BBZ and Pep42-BBZ CAR-T cell treatment groups demonstrated that CSCs marked by CD133 were significantly wounded in vivo. Upon imaging, we found that all but one mouse showed a shrinkage of their tumors 14 days following injection with Pep42-BBZ CAR-T cells. We studied three mice further, for up to 63 days, and found no evidence of relapse. These data suggested that Pep42 CAR-T cells efficiently killed both A549 tumor cells and derived CSCs both in vitro and in vivo, which ultimately resulted into the elimination of tumor xenografts.

In some cases of its application, CAR-T cell therapy has been reported to result in major treatment-related toxicities, including cytokine release syndrome (CRS) and immune effector cell-associated neurotoxicity syndrome (ICANS) [49]. Both CRS and ICANS are caused by the activation of CAR-T cells and cytokines secreted by the associated immune cells [50]. However, we found little evidence for significant off-target toxicity to healthy tissues [51] of our Pep42 CAR-T cells, as confirmed after examining multiple organs from multiple treated mice in our study. Also, given that the coding sequence of *Mus musculus* GRP78 (used in our study) is highly homologous to its ortholog in *Homo sapiens*, we postulate that our mouse studies can reliably guide the evaluation of the safety profile for designs of csGRP78-targeting CAR-T cell therapies that are effective for humans. As shown in our H&E staining studies, we did not observe significant tissue injury in multiple organs examined 5 days after intravenous injection of CAR-T cells. Furthermore, for mice injected with Pep42-BBZ CAR-T cells, there was some evidence of T cells within the vessel cavities of the lung and liver, as expected, while a significant T cell accumulation was documented at tumor sites. Based on our results, we conclude that Pep42-BBZ CAR-T cells are effective for the in vitro and in vivo killing of non-small-cell lung carcinoma cells that express csGRP78. As CAR-T cells are currently under investigation, as mentioned above, though MSLN and PD-1 are both kinds of consistent cell-surface proteins at a relatively low level in normal tissues, they are not tumor-specific antigens. As a result, off-target toxicity will occur under circumstances. However, the translocation in cancer cells endows csGRP78 with the characteristics of a tumor-specific antigen to some extent. Therefore, we speculate that csGRP78-targeted CAR-T cells will be superior to other targeted CAR-T cells in terms of off-target toxicity. However, the biology, physiology, and immunology of mice are quite different from those of humans. To confirm the safety of this CAR-T cell more accurately, further work like a safety evaluation in nonhuman primates will be necessary. The physiology and immunity of nonhuman primates is closest to those of humans; this will reveal the impact on patients, which is required for clinical translation.

## 4. Materials and Methods

### 4.1. Cell Lines and Culture

Two non-small-cell lung cancer (NSCLC) cell lines, A549 and H1299, and 293T cells were purchased from the Chinese National Infrastructure of Cell Line Resources (Kunming, China). Cells were cultured in a complete medium composed of DMEM with 10% FBS, 100 U/mL penicillin, and 100 mg/mL streptomycin (Life Technologies, Carlsbad, CA, USA). A549- or H1299-derived cancer stemlike cells (CSCs), named A549 CSCs or H1299 CSCs, were cultured with a conditioned stem-cell medium of serum-free DMED/F12, supplemented with 20 ng/mL EGF, 20 ng/mL bFGF and 1× B27 (all supplements purchased from Life Technologies, Carlsbad, CA, USA). All cells were grown in a humidified incubator with 5% CO_2_ at 37 °C.

### 4.2. Plasmid Design and Lentivirus Package

A csGRP78-targeting CAR construct, named Pep42-BBZ, was prepared as previously described [31]. The peptide Pep42 was synthesized (BGI, Beijing, China) and cloned into a second-generation CAR lentiviral vector, which encoded a CD8 hinge spacer, a CD8 transmembrane domain, a costimulatory signal 4-1BB, and a CD3ζ endo-domain, fused with the coding sequence for the far-red fluorescence marker protein mkate2 through a self-cleaving T2A linker sequence. A negative control mock-BBZ construct was prepared using the same CAR lentiviral vector construct without the Pep42 sequence. To prepare Pep42-BBZ or mock-BBZ lentivirus, each plasmid was cotransduced with the lentiviral packaging plasmids pMD2.G and pCMVΔ8.91 (Addgene, Watertown, MA, USA) into HEK293T cells at a ratio of 20:4:10. Following 48 h and 72 h from transfection, supernatants containing the lentivirus were collected for concentration by centrifugation and titration for later use.

### 4.3. Production of CAR-T Cells

T cells were derived from the peripheral blood of healthy donors and purified by the RosetteSep™ Human T Cell Enrichment Cocktail (STEMCELL, Vancouver, Canada). Cells were cultured in advanced 1640 medium (Life Technologies, Carlsbad, CA, USA) supplemented with 10% FBS, 100 U/mL penicillin, 100 mg/mL streptomycin, 1× Glutamax (Gibco Life Technologies, Carlsbad, CA, USA), and 200 U/mL human IL-2 (PeproTech, Cranbury, NJ, USA) and mixed with CD3/CD28 Dynabeads (Life Technologies, USA). After activated by beads for 72 h, T cells were then transduced with either Pep42-BBZ or mock-BBZ plasmid-containing lentivirus, at a MOI of 30 with the addition of the transfection reagent lentiboost (SIRION biotech, Martinsried, Germany) for 24 h. Following another 96 h of culturing, three groups of CAR-T cells were processed for the CAR expression (mkate2 ratio) test, T cell phenotyping evaluation using flow cytometry, and further experimentations. For injection into mice, Dynabeads were removed with magnetic separation.

### 4.4. T Cell Phenotyping

Flow cytometry was performed to assess T cell phenotypes using the following antibodies: FITC anti-CD4 antibody (357406, BioLegend, San Diego, CA, USA), APC-Cy7 anti-CD8 antibody (344714, BioLegend), PE anti-CD45RO antibody (304206, BioLegend), and APC anti-CCR7 (353214, BioLegend). Stained cells were tested by flow cytometry in a staining buffer (phosphate-buffered saline (PBS) with 2% fetal bovine serum). Data were analyzed using Flowjo V10 software.

### 4.5. Immunofluorescence

A549 CSCs and H1299 CSCs were allowed to settle onto coverslips precoated with laminin (Gibco, USA) for 24 h in advance. Adherent cultures of A549 and H1299 cells, as well as the two CSCs were established in plates at a seeding density of 6 × 10^5^/mL. At the end of the culture period, cells were fixed onto coverslips and processed for immunostaining, as follows. Cells were gently washed with PBS for 5 min and incubated with a solution of anti-GRP78 antibody (PA1-014A, Invitrogen, Grand Island, NY, USA) at a 1:200 dilution for 1 h at room temperature. Following three PBST washes of 5 min, cells were fixed by 4% paraformaldehyde for 15 min and blocked with a solution of 10% goat serum in 0.1% BSA, 0.1% Tween-20, and PBS for 1 h. Then, cells were stained with a Cy3-labeled goat antirabbit antibody (A10520, Invitrogen) at a 1:1000 dilution for 1 h at room temperature in the dark and washed with PBST three times. Next, cells were once again blocked for 1 h and stained with anti-β-catenin antibody (ET1601-5, Huabio, Hangzhou, China) at a 1:100 dilution at 4 °C in the dark overnight. After three PBST washes, they were stained with the Alexa Flour 488-labeled goat antirabbit IgG antibody (A11008, Invitrogen) at a 1:1000 dilution in the same condition as with the above secondary antibody. After another three PBST washes for 5 min each time, cells were stained with 5 μg/mL DAPI for 10 min in the dark. Finally, adherent cells on the coverslip were mounted on a glass slide using mounting medium (Polysciences, Warrington, PA, USA), allowed to dry briefly, and then stored at 4 °C, with care taken to avoid light exposure. Negative control (CON) experiments were performed in which cells on coverslips were incubated without the primary antibody, so as to confirm immunolabelling specificity.

### 4.6. Cytotoxicity Assays In Vitro

To determine the cytotoxicity of CAR-T cells to A549, H1299, A549 CSCs, and H1299 CSCs, a luciferase-based assay was used. A549 cells and H1299 cells were transduced with lentivirus-containing luciferase plasmids and cultured to cultivate luciferase-expressing cell lines, A549-luc and H1299-luc. A549 CSC-luc and H1299 CSC-luc were cultured from A549-luc and H1299-luc, respectively. Prior to subjecting the CSCs to a CAR-T cell cytotoxicity assay, they were adhered to the bottom of a culture plate coated with laminin (Gibco Life Technologies, Carlsbad, CA, USA) and allowed to settle for 12 h before experimentation. In total, 2 × 10^3^ target cells were seeded in each well of a 96-well plate. Nontransduced (NTD), mock-BBZ, and Pep42-BBZ CAR-T cells were, respectively, cocultured with target cells in 96-well plates at the effector cell: target cell (E:T) ratio = 1:1, 2:1, 4:1, or 8:1. After 20 h, the supernatant was removed and stored at −80 °C for subsequent ELISA assays. Cells were lysed, and 30 μL of luciferase substrate (Promega, Madison, WI, USA) was added. Luminescence was measured on a microplate reader. All cytotoxicity values were calculated based on NTD cytotoxicity at the corresponding E:T ratios.

### 4.7. ELISA

Measurements of the levels of IFN-γ in supernatants collected from cytotoxicity experiments were assessed by enzyme-linked immunosorbent assay kits (Invitrogen, Grand Island, NY, USA) following the manufacturer’s instructions.

### 4.8. Xenograft Mouse Model

All protocols were approved by the animal ethics committee of the West China Hospital, Sichuan University. Female severely immune-deficient NOD-Prkdcem26Cd52Il2rgem26Cd22/Nju (NCG, T001475) mice purchased from the GemPharmatech Co., Ltd. of Nanjing (China) were 6 to 8 weeks old and housed in a specific pathogen-free environment, within the Laboratory Animal Center of West China Hospital. For each mouse, an injection of 100 μL of A549-luc cell suspension, containing 5 × 10^5^ cells, 0.9% saline, and 30% Matrigel (BD Bioscience, San Jose, CA, USA) was made subcutaneously into the right flank. Five days later, mice were numbered and divided into three groups by a random-number-drawing method: (1) control group: nontransduced (NTD) T cell group with 5 mice; (2) vector control group: mock-BBZ CAR-T cell group with 6 mice; (3) Pep42-BBZ CAR-T cell group with 6 mice. Next, each mouse from each of the three groups received corresponding T cell injections delivered intravenously through the tail vein, as follows: 1 × 10^7^ NTD T cells, 1 × 10^7^ mock-BBZ CAR-T cells, and 1 × 10^7^ Pep42-BBZ CAR-T cells. Bioluminescent signals were monitored once a week using an in vivo imaging software (IVIS) system (Lumina Xr) and quantified by Living Image Software version 4.2 (Caliper Life Science, Hopkinton, MA, USA). The diameter of tumors was calibrated every 2–3 days using a vernier caliper and the volume of tumors was calculated according to the formula: tumor volume = 1/2 × (short diameter)^2^ × long diameter. To ensure blinding during tumor measurement, the measurement operator did not know which group the measured mice came from, while the other recorder knew. When the tumor volume reached approximately 2000 mm^3^, mice were euthanized.

### 4.9. H&E Staining and Immunofluorescence Analysis

Hematoxylin and eosin (H&E) staining of sections of mouse heart, liver, spleen, lung, kidney, and brain samples was conducted as follows. Organs were first dissected and fixed in 4% neutral formalin for 72 h. The tissues were subsequently embedded in paraffin and sectioned using a microtome to prepare sections of 4 μm thickness, before the sections were floated onto glass slides and allowed to dry briefly. Next, the sections on slides were deparaffinized, rehydrated, and subjected to hematoxylin and eosin stain before being mounted using coverslips. For immunofluorescence (IF) staining experiments, the rehydrated tumor, liver, and lung tissue sections were processed for citrate-antigen retrieval. Following this step, sections were washed in PBST and briefly incubated with a solution of 10% serum to block nonspecific epitopes. Then, the appropriate primary antibodies including anti-GRP78 antibody (PA1-014A, Invitrogen), anti-CD3ζ antibody (ET1607-20, Huabio, Hangzhou, China), anti-CD133 antibody (66666-1-Ig, proteintech, Wuhan, China), anti-ki67 antibody (ab16667, abcam, Cambridge, UK), or anticleaved caspase-3 antibody (9661, Cell Signaling Technology, Danvers, MA, USA) were, respectively, applied and incubated with the sections overnight at 4 °C. The next day, the slides were rinsed with PBST three times (5 min/time) and secondary antibodies including Cy3-conjuncted goat antirabbit IgG (A10520, Invitrogen) or goat antimouse IgG (A10521, Invitrogen) were incubated with the sections for 1 h at room temperature away from the light. Later, sections were washed with PBST three times (5 min/time) and stained with DAPI to label nuclei. Finally, the sections on slides were mounted with a mounting medium (AB104135, Abcam, Cambridge, UK) and covered with coverslips before microscopy was carried out to visualize the immunostained markers.

### 4.10. RNA Extraction and Quantitative Real-Time PCR

RNA was extracted from the A549 cell line and A549 CSCs as described previously [52]. The stem-cell markers CD133, SOX2, and Nanog were detected using an SYBR Green QRT-PCR Kit (A25742, Thermo Fisher, Waltham, MA, USA). QRT-PCR was performed using the following conditions: 2 min for 50 °C, 10 min for 95 °C, followed by 40 cycles of denaturation at 15 s for 95 °C and annealing/extension of 1 min for 60 °C. 18S rRNA was used as an internal standard. The primers used were as follows:

CD133 F: 5’-AGTCGGAAACTGGCAGATAGC-3’, 

CD133 R: 5’-GGTAGTGTTGTACTGGGCCAAT-3’,

SOX2 F: 5’-CTCGTGCAGTTCTACTCGTCG-3’, 

SOX2 R: 5’-AGCTCTCGGTCAGGTCCTTT-3’,

Nanog F: 5’-TTTGTGGGCCTGAAGAAAACT-3’, 

Nanog R: 5’-AGGGCTGTCCTGAATAAGCAG-3’,

18s F: 5’-GTAACCCGTTGAACCCCATT-3’, 

18s R: 5’-CCATCCAATCGGTAGTAGCG-3’.

### 4.11. Statistical Analysis

All statistics were analyzed using GraphPad Prism 7.0 statistical software. Data were presented as mean ± SD. Statistical differences between two groups were analyzed using Student t tests with Welch’s correction. Statistical differences among three or more groups were analyzed by one-way ANOVA comparisons. Statistical significance was defined as * *p* ≤ 0.05, ** *p* ≤ 0.01, and *** *p* ≤ 0.001.

## 5. Conclusions

In conclusion, our study showed that csGRP78 was expressed on two lung cancer cell lines and the derived CSCs, and the treatment of these cancer cells with csGRP78-targeting Pep42 CAR-T cells led to their cytotoxic killing in vitro. We also found that Pep42 CAR-T cells effectively shrank lung tumor xenografts derived from A549 tumor cells with no overt off-target effects on multiple organs of the body. Our studies suggest that csGRP78 may be a viable tumor target to develop effective CAR-T cell therapies to treat human lung cancer.

## Data Availability

The datasets of this work are available from the corresponding author on reasonable request.

## Supplementary Materials

The following supporting information can be downloaded at: https://www.mdpi.com/article/10.3390/ijms25010564/s1.

## Abbreviations

CAR-T cells = chimeric antigen receptor-T cells; csGRP78 = cell-surface glucose-regulated protein 78; ER = endoplasmic reticulum; IF = immunofluorescence; CSCs = cancer stem cells; IFN-γ = interferon γ; NSCLC = non-small-cell lung cancer; EGFR = epidermal growth factor receptor; MSLN = mesothelin; PD-L1 = programmed cell death ligand 1; B7-H3 = B7 homolog 3; CEA = carcinoembryonic antigen; CSCs = cancer stem cells; NTD = nontransduced; IVIS = in vivo imaging software; CRS = cytokine release syndrome; ICANS = immune effector cell-associated neurotoxicity syndrome.
