# Supplementary figures and images for: Cell-Surface GRP78-Targeted Chimeric Antigen Receptor T Cells Eliminate Lung Cancer Tumor Xenografts
Source: Int J Mol Sci. 2024 Jan 1;25(1):564. doi: 10.3390/ijms25010564 (PMC10779323; doi:10.3390/ijms25010564)

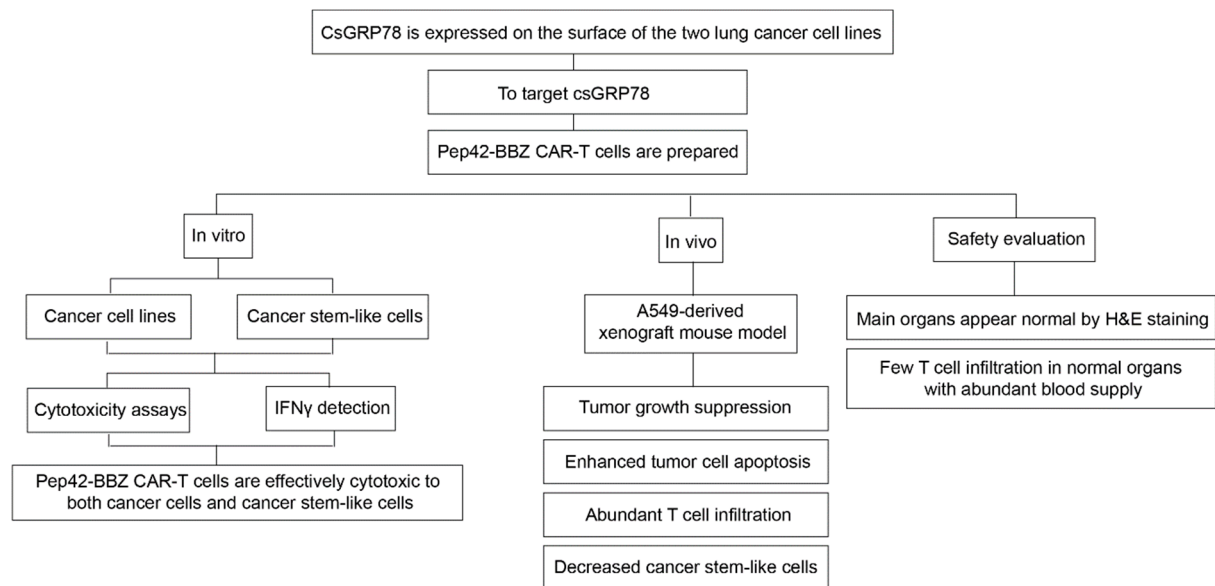

Supplementary Figure S1. Schematic diagram of the overall study design and main conclusions.

Supplement: Supplementary file 1 [file ijms-25-00564-s001.zip › Supplementary Figure S1.pdf]
